# Microbial community structure in a biogas digester utilizing the marine energy crop *Saccharina latissima*

**DOI:** 10.1007/s13205-012-0097-x

**Published:** 2012-10-25

**Authors:** Phillip B. Pope, Vivekanand Vivekanand, Vincent G. H. Eijsink, Svein J. Horn

**Affiliations:** Department of Chemistry, Biotechnology and Food Science, Norwegian University of Life Sciences, Post Office Box 5003, 1432 Ås, Norway

**Keywords:** Biogas, Anaerobic digestion, Seaweed, Macroalgae, Methane

## Abstract

Seaweed is a highly attractive marine crop for the production of biofuels, due to its rapid growth rate as well as high polysaccharide and low lignin content. One appealing exploitation route is the production of biogas by anaerobic digestion. Interestingly, despite the compositional differences between seaweed and lignocellulosic biomass, available data indicate that conditions and inocula traditionally used for the latter may work well for seaweed. To gain more insight into the underlying microbial processes, we have generated 16S rRNA gene amplicon pyrosequencing data to comparatively describe microbial communities in biogas digesters containing either the seaweed *Saccharina latissima* or wheat straw. The seaweed digesters gave better biogas yield and a higher relative abundance of core group *Methanosaeta*-affiliated *Archaea*. Conversely, variation in biomass had only minor abundance effects towards dominant bacterial lineages and influenced only low-abundant bacterial OTUs. Affiliations between dominant archaeal and bacterial phylotypes described here and previously identified anaerobic digestion core groups indicate that trends are beginning to emerge within these newly explored microbial ecosystems, the understanding of which is currently impeded by limited published datasets.

Biogas production, particularly in the purified form of biomethane, is seen as a vital component of renewable energy technologies due to the wide variety of organic sources that can be used and the compatibility of methane with existing energy infrastructure. Efforts to augment the biogas processes have focused on utilizing waste materials as well as alternative biomass substrates that lessen the impact on arable land. To that end, seaweed species have been identified as high potential substrates for biomethane production, due to their rapid growth rate as well as high polysaccharide (~60 %) and low lignin content (Horn 2009). Compared to organic waste streams and terrestrial biomasses, relative little is known on the anaerobic digestion (AD) of marine substrates. Available data for seaweed are, however, quite promising, in particular for the brown seaweed *Saccharina latissima* (Nielsen and Heiske 2011; Vivekanand et al. 2012). Seaweed is less recalcitrant than lignocellulosic materials meaning that thermal pretreatments that are often used to speed up biogas processes can be milder, thus reducing the risk of inhibitor formation that is common during the harsher pretreatments (Vivekanand et al. 2012).

In this study, we report compositional and comparative analysis of the microbial communities in anaerobic digesters. 16S rRNA gene amplification for both bacterial and archaeal domains was performed to ensure that representatives for all the key metabolic stages of AD were enveloped, that is, polymer hydrolysis, sugar fermentation, acetogenesis (all *Bacteria*) and methanogenesis (*Archaea*). Samples were collected from three 1.1 L batch digesters run in triplicate for 119 days at 37 °C (stable pH 7.3 over the course of the experiment). All three digesters were inoculated with 600 mL of pre-incubated waste water sludge containing 10.5 g L^−1^ of volatile solids (VS) (Vivekanand et al. 2012), and were defined according to the new substrate added (1.05 g VS added at day 0 and 67, 2.1 g VS total): inoculum containing no additional organic substrate (IC), inoculum with seaweed (*S. latissima*, IC + SW) and inoculum with steam exploded wheat straw (*Triticum aestivum*, IC + WS). The total liquid volume in all digesters was then adjusted to 700 mL by adding distilled water. Total methane production in IC + SW (223 ±  61 mL g^−1^ VS) was approximately twice as high as in IC + WS (98 ±  44 mL g^−1^ VS); note that SW and WS materials have different C/N ratios of 8.8 and 98.4, respectively (Vivekanand et al. 2012). For each digester, sub-samples from each triplicate (equal volume) were pooled, and DNA extraction was performed as described by Rosewarne et al. (2010). *Rrs* genes were amplified using the broadly conserved primer sets 27F-515R [*Bacteria*: (Pope et al. 2012)] and 340F-1000R [*Archaea*: (Gantner et al. 2011)], both containing the 454 Life Sciences primer A sequence and a unique 8-nt multiplex identifier (Hamady et al. 2008). *Rrs* gene sequences were quality filtered using the QIIME software package (Caporaso et al. 2010), whilst error correction and chimera removal were performed using OTUPIPE which incorporates UCHIME (Edgar et al. 2011). Operational taxonomic units (OTUs) were clustered at 97 % sequence identity using UCLUST software (Edgar 2010) and taxonomy was assigned using the Ribosomal Database Project classifier (Cole et al. 2003). After filtering and normalization (datasets randomly “subsampled” to remove sample heterogeneity), 1,992 bacterial and 651 archaeal 16S rRNA sequences (in total) clustered into 63 and 14 OTUs, respectively (Table 1; Acc. Numbers JX279942–JX280018). Rarefaction analysis showed that the three digester datasets afforded a similar degree of adequate coverage of bacterial biodiversity within each digester (Fig. 1; Table 1). Moreover, Fig. 1 illustrated that the addition of seaweed appears to reduce archaeal species diversity.

**Table 1:** Archaeal (ARC) and bacterial (BAC) operational taxonomic unit (OTU) representatives of *rrs* gene sequences obtained from biogas digesters containing waste water sludge as inoculum (IC), IC plus wheat straw (IC + WS) or IC plus seaweed (IC + SW)

ID	IC	IC + WS	IC + SW	Consensus Lineage^a^	Cult_rep (Acc. number)	ID (%)	Clone_rep_env (Acc. number)	ID (%)
ARC-1	32	103	142	o_*Methanosarcinales*	*Methanosaeta concilii* (X16932)	98	WWS (CU916103) Core Gp. VI^b^	99
ARC-2	87	51	40	o_*Methanomicrobiales*	*Methanosphaerula palustris* str. E1-9c (EU156000)	96	Sediment enrichment (FR845732)	99
ARC-3	47	9	13	o_*MethanoBacteriales*	*Methanobacterium alcaliphilum* (AB496639)	85	WWS (CU917028) Core Gp. V^b^	99
ARC-4	13	11	5	o_*Methanomicrobiales*	*Methanospirillum hungatei* str. JF-1 (CP000254)	99	UASB reactor (EU888810)	99
ARC-5	17	14	6	o_*Methanomicrobiales*	*Methanospirillum* sp. (AJ133792)	99	WWS (CU916087)	99
ARC-6	1	4	2	p_*Crenarchaeota*	*Candidatus Nitrososphaera gargensis* (EU281332)	84	WWS (CU915923)	99
ARC-7	1	2	0	o_*Methanomicrobiales*	*Methanoculleus* sp. LH2 (DQ987521)	92	WWS (CU915985)	95
ARC-8	3	3	0	o_*Methanomicrobiales*	*Methanospirillum* sp. (AJ133792)	97	WWS (CU917418) Core Gp. III^b^	99
ARC-9	2	0	0	o_*MethanoBacteriales*	*Methanobacteriumferruginis* (AB542743)	99	Oil reservoir (HQ395111)	99
ARC-10	0	4	0	o_*Methanosarcinales*	*Methanosaeta concilii* (X16932)	95	WWS (CU915904)	97
ARC-11	1	3	1	o_*Methanomicrobiales*	*Methanosphaerula palustris* str. E1-9c (EU156000)	94	WWS (CU917018)	97
ARC-12	2	1	0	o_*Methanomicrobiales*	*Methanosphaerula palustris* str. E1-9c (EU156000)	94	Biogas Plant (EU857631)	99
ARC-13	3	0	0	o_*MethanoBacteriales*	*Methanolineatarda* str. NOBI-1 (AB162774)	84	WWS (CU916898)	97
ARC-14	0	4	0	o_*Methanomicrobiale*	*Methanospirillum hungatei* (M60880)	95	Sediment enrichment (FR845732)	96
BAC-1	246	118	215	p_*Spirochaetes*; f_*Spirochaetaceae*	*Treponema primitia* str. ZAS-1 (AF093251)	86	WWS (CU922923)	99
BAC-2	126	147	68	k_*Bacteria*	*Citricoccus muralis* str. 4-0 (AJ344143)	77	WWS (JQ157767)	99
BAC-3	94	88	100	p_*Bacteroidetes*; o_*Bacteroidales*	*Alistipes shahii* str. JCM 16773 (AB554233)	79	WWS (CU922937)	99
BAC-4	25	61	51	p_*Chloroflexi*; f_*Anaerolinaceae*	*Thermoanaerobacterium thermosaccharolyticum* (EU563362)	80	WWS (CU918793) Core Gp. III^b^	99
BAC-5	22	24	19	p_*Chloroflexi*; f_*Anaerolinaceae*	*Clostridium* sp. str. RPec1 (Y15985)	76	WWS (CU920051) Core Gp. VI^b^	99
BAC-6	16	19	19	k_*Bacteria*	*Brevibacillus invocatus* str. 1P02AnA (EU977716)	82	Oil-cont. soil	99
BAC-7	10	18	40	p_*Bacteroidetes*; f_*Flammeovirgaceae*	*Mucilaginibacter* sp. str. DR-f1 (GU139694)	82	WWS (JQ106146)	98
BAC-8	25	21	14	p_*Spirochaetes*; f_*Spirochaetaceae*	*Treponema primitia* str. ZAS-1 (AF093251)	86	WWS (JQ346773)	99
BAC-9	5	22	19	p_*Bacteroidetes*; f_*Porphyromonadaceae*	*Bacteroides* sp. str. SA-11 (AY695842)	90	BR-thermophilic (FN436125)	99
BAC-10	10	28	5	p_*Actinobacteria*; c_*Actinobacteria*	*Geobacillus thermocatenulatus* hs6 (AY550104)	84	Oil-cont. soil (HQ689298)	98
BAC-11	2	11	4	k_*Bacteria*	*Saccharococcus thermophilus* str. ATCC 43125 (X70430)	75	WWS (JQ098865)	99
BAC-12	2	3	16	p_*Bacteroidetes*; o_*Bacteroidales*	*Bacteroides* sp. str. 3_1_19 (ADCJ01000062)	80	WWS (JQ124386)	99
BAC-13	7	7	0	p_*Proteobacteria*; f_*Hydrogenophilaceae*	*Petrobacter succinatimandens* str. BON4 (AY219713)	99	WWS (AF280851)	99
BAC-14	4	3	3	p_*Actinobacteria*; c_*Actinobacteria*	*Bacillus* sp. str. BR (AM050346)	74	WWS (CU917482)	99
BAC-15	0	3	2	p_*Synergistetes*; f_*Anaerobaculaceae*	*Thermovirga lienii* str. Cas60314 (DQ071273)	87	BR-Low temp. (FJ164079)	99
BAC-16	2	5	4	p_*Chloroflexi*; f_*Anaerolinaceae*	*Dehalogenimonas lykanthroporepellens* (CP002084)	75	Sulfate-reducing bioreactor (DQ443984)	97
BAC-17	1	5	3	p_*Acidobacteria*; o_*Acidobacteria*les	*Holophaga foetida* (X77215)	80	Oxic rice field soil (AY360604)	99
BAC-18	1	6	2	p_*Spirochaetes*; f_*Spirochaetaceae*	*Spirochaeta stenostrepta* str. JCM 16534 (AB541984)	97	MFC rice (GQ458085)	99
BAC-19	2	4	4	p_*Bacteroidetes*; o_*Bacteroidales*	*Eubacterium* sp. str. F1 (EU281854)	82	WWS (CU918036)	99
BAC-20	7	1	1	p_*Spirochaetes*	*Treponema primitia* str. ZAS-1 (AF093251)	83	WWS (JQ106578)	98
BAC-21	0	3	6	k_*Bacteria*	*Spirochaeta xylanolyticus* (AY735097)	78	WWS (JQ118642)	99
BAC-22	4	5	3	k_*Bacteria*	*Citricoccus* sp. str. 3056 (AM111007)	75	WWS (JQ136258)	99
BAC-23	0	5	0	k_*Bacteria*	*Streptomyces* sp. str. 21-4 (AB222072)	77	WWS (JQ096165)	99
BAC-24	1	4	4	p_*Bacteroidetes*; f_*Porphyromonadaceae*	*Bacteroides* sp. str. SA-7 (AY695838)	88	WWS (CU920278)	99
BAC-25	1	0	0	p_*Spirochaetes*; f_*Spirochaetaceae*	*Spirochaeta xylanolyticus* (AY735097)	85	WWS (JQ159995)	98
BAC-26	0	0	9	p_*Lentisphaerae*; f_*Victivallaceae*	*Victivallis vadensis* str. ATCC BAA-548 (NR_027565)	94	MFC palm oil mill effluent (JF309189)	99
BAC-27	2	1	2	p_*Spirochaetes*	*Spirochaeta xylanolyticus* (AY735097)	85	WWS (JQ158980)	98
BAC-28	1	3	4	p_*Firmicutes*; c_*Clostridia*	*Caloramator* sp. str. 8 (EU621406)	84	Food-processing wastes (GU389808)	98
BAC-29	1	2	4	p_*Spirochaetes*; f_*Spirochaetaceae*	*Spirochaeta zuelzerae* (M88725)	92	WWS (JQ111324)	99
BAC-30	2	0	4	p_OP8	*Geobacillus thermodenitrificans* str. BGSC 94A1 (AY608960)	79	WWS (GQ480154)	99
BAC-31	3	1	0	p_WS1	*Streptomyces scabrisporus* (EU841700)	78	WWS (CU917740)	99
BAC-32	3	1	3	k_*Bacteria*	*Moorella thermoacetica* str. DSM 7417 (FJ888654)	82	WWS (JQ096458)	98
BAC-33	1	3	0	p_*Chloroflexi*; f_*Anaerolinaceae*	*Thermodesulfobium narugens* (AB077817)	80	WWS (CU927349)	99
BAC-34	0	3	1	p_*Bacteroidetes*; o_*Bacteroidales*	*Persicivirga* sp. str. PHSCD-1 (HM854017)	80	WWS (JQ127396)	99
BAC-35	3	1	1	p_*Spirochaetes*; f_*Spirochaetaceae*	*Spirochaeta xylanolyticus* (AY735097)	85	WWS (JQ091697)	99
BAC-36	1	2	3	p_*Synergistetes*; f_*Synergistaceae*	*Synergistes* sp. str. RMA 16088 (DQ412718)	89	BR (EF583500)	99
BAC-37	1	0	2	p_*Proteobacteria*; f_*Syntrophaceae*	*Smithella propionica* str. LYP (AF126282)	89	WWS (JQ099713)	99
BAC-38	2	2	0	p_*Proteobacteria*; f_*Syntrophorhabdaceae*	*Myxococcus fulvus* str. 0198-1 (EU263001)	80	Petroleum reservoir (JN627945)	99
BAC-39	1	2	2	p_WS1	*Streptacidiphilus* sp. str. Aac-20 (AB180766)	79	WWS (JQ141219)	99
BAC-40	3	4	0	p_*Firmicutes*; c_*Clostridia*	*Caloramator* sp. str. 8 (EU621406)	86	WWS (CU921657)	99
BAC-41	0	4	2	p_SAR406	*Desulfuromonas acetexigens* (U23140)	80	WWS (CU922995)	99
BAC-42	1	0	2	p_*Spirochaetes*; f_*Spirochaetaceae*	*Spirochaeta xylanolyticus* (AY735097)	85	WWS (JQ158980)	99
BAC-43	2	0	1	p_*Proteobacteria*; f_*Comamonadaceae*	*Acidovorax* str. R-25075 (AM084109)	98	Freshwater spring (AB425064)	99
BAC-44	0	4	1	p_*Chloroflexi*; f_*Anaerolinaceae*	*Clostridium proteolyticum* str. DSM 3090 (X73448)	75	WWS (CU921614)	99
BAC-45	2	1	2	p_*Chloroflexi*; f_*Anaerolinaceae*	*Clostridium botulinum* str. ATCC 19397 (CP000726)	77	Anaerobic swine lagoon (AY953235)	97
BAC-46	0	4	1	p_*Chloroflexi*; f_*Anaerolinaceae*	*Clostridium difficile* str. 630 (NC_009089)	74	WWS (JQ137633)	99
BAC-47	0	0	5	p_*Spirochaetes*; f_*Spirochaetaceae*	*Spirochaeta stenostrepta* str. JCM 16534 (AB541984)	89	WWS (JQ346773)	99
BAC-48	1	2	0	p_*Spirochaetes*; f_*Spirochaetaceae*	*Treponema primitia* str. ZAS-1 (AF093251)	89	WWS (JQ346773)	99
BAC-49	0	0	4	p_*Proteobacteria*; f_*Desulfovibrionaceae*	*Desulfovibrio* str. Ds3 (EU326029)	99	BR-carrot waste (JF533850)	99
BAC-50	5	0	0	p_*Spirochaetes*; f_*Spirochaetaceae*	*Spirochaeta xylanolyticus* (AY735097)	85	WWS (JQ158980)	99
BAC-51	4	0	0	k_*Bacteria*	*Spirochaeta xylanolyticus* (AY735097)	86	Oil-cont. soil (HQ689200)	95
BAC-52	2	1	0	p_*Bacteroidetes*; o_*Bacteroidales*	*Capnocytophaga canimorsus* str. CIP 103936 (AY643075)	88	BR-refuse (GQ453634)	94
BAC-53	0	2	1	k_*Bacteria*	*Bacillus* sp. str. JS4 (AY372924)	83	WWS (JQ144546)	100
BAC-54	0	4	1	p_*Chloroflexi*; f_*Anaerolinaceae*	*Thermodesulfobium narugense* DSM 14796 (NR_024789)	77	WWS (CU918060)	99
BAC-55	2	0	0	p_*Actinobacteria*; o_Corio*Bacteria*les	*Streptomyces* sp. str. Z61 (EF012131)	85	Natural gas enrichment (EU037971)	99
BAC-56	0	1	2	p_*Armatimonadetes*	*Symbiobacterium thermophilum* str. IAM 14863 (NC_006177)	76	Microbial mat (FJ207112)	84
BAC-57	1	0	0	p_*Proteobacteria*; f_*Syntrophobacteraceae*	*Syntrophobacter sulfatereducens* str. TB8106 (AY651787)	99	WWS (CU923992)	99
BAC-58	1	0	2	p_WS1	*Thermoactinomyces sacchari* str. KCTC 9790 (AF138737)	81	BR-brewery waste (EF515625)	99
BAC-59	2	1	0	p_*Bacteroidetes*; o_*Bacteroidales*	*Persicivirga* sp. str. PHSCD-1 (HM854017)	79	Waste silk refining system (HQ453334)	98
BAC-60	0	1	2	k_*Bacteria*	*Alistipes putredinis* str. ATCC 29800 (NR_025909)	76	WWS (JQ093377)	95
BAC-61	2	0	1	p_*Proteobacteria*; c_Beta*Proteobacteria*	*Azonexus* sp. str. HME6654 (HM590828)	99	WWS (JQ413515)	99
BAC-62	2	0	0	p_*Proteobacteria*; f_Rhodocyclaceae	*Rhodocyclus* sp. str. HOD 5 (AY691423)	96	WWS (JQ177298)	98
BAC-63	0	3	0	p_*Proteobacteria*; f_Comamonadaceae	*Acidovorax* sp. str. GPTSA100-27 (DQ854967)	94	Activated sludge (EU104267)	97

^a^Hierarchical taxonomic assignment for each OTU calculated using the RDP naïve Bayesian Classifier (Cole et al. 2003). Deepest lineage assignments (*k* kingdom, *p* phylum, *c* class, *o* order, *f* family) are displayed only where OTUs could be assigned with an 80 % bootstrap confidence estimate

^b^Indicates affiliation to highly prevalent core phylotypes involved in AD of sludge that were previously described in (Rivière et al. 2009)

*BR* biogas reactor, *WWS* waste water sludge, *MFC* microbial fuel cell

**Fig. 1:**
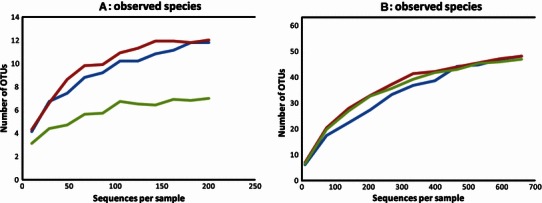
Rarefaction analyses using operational taxonomic unit (OTU) frequency of archaeal (**a)** and bacterial (**b)***rrs* gene datasets obtained from the biogas digesters containing waste water sludge as inoculum (IC *blue*), IC plus wheat straw (IC + WS *red*) or IC plus seaweed (IC + SW *green*). A 97 % sequence identity threshold has been employed for the OTU constructions used in these analyses

Comparisons of the archaeal communities revealed that OTUs ARC_nor-1, ARC_nor-2 and ARC_nor-3, affiliated to the Taxonomic Order-ranks *Methanosarcinales*, *Methanomicrobiales* and *Methanobacteriales*, respectively, were dominant in all three samples (Fig. 2a–b). However, their composition varied considerably depending on the digester substrate (Fig. 2b–c). The increased dominance of ARC_nor-1 in the IC + SW digester coincided with higher methane production [Fig. 2; (Vivekanand et al. 2012)], as well as a slightly higher methane content in the biogas (57 % vs. 53 % in IC + WS). Affiliation of ARC_nor-1 to an acetoclastic methanogen (*Methanosaeta concilii*; 98 % ID) was also in agreement with *Methanosaeta* dominance in AD communities that utilize freshwater algae substrates (Ellis et al. 2012). In contrast, hydrogenotrophic methanogens, of which ARC_nor-2 is putatively categorized, were most dominant in the inoculum digester (IC), and their relative abundance decreased in digesters containing either IC + WS or IC + SW (Fig. 2b). Interestingly, both ARC_nor-1 and ARC_nor-3 were affiliated (99 % ID; Table 1) to previously described and repeatedly detected core group phylotypes (OTU-VI and OTU-V, respectively), which dominate sludge AD communities (Rivière et al. 2009).

**Fig. 2:**
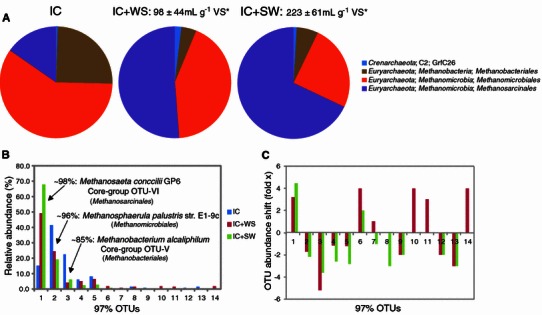
Relative abundance and comparison profiles of archaeal 16S rRNA OTUs identified in anaerobic digesters containing either waste water sludge with no additional organic substrate (inoculum, IC), IC plus wheat straw (IC + WS), or IC plus seaweed (IC + SW). **a**, **b** The relative abundance of archaeal lineages at a phylum-level and OTU-level, respectively. OTU abundance shifts between WS and SW digesters **c** were measured as either fold-change increases (+) or decreases (−) against IC measurements. Colour coding in **b** and **c** are as follows: *blue* indicates IC, *red* indicates IC + WS and *green* indicates IC + SW. Lineage information for selected OTUs and OTU affiliation to previously described, highly prevalent core phylotypes (Rivière et al. 2009) is provided. OTUs numbers in the *x*-axis correspond to ARC_nor-terminology referred to in the text. Total methane yields are included in **a** for IC + WS and IC + SW, which are provided in the original publication on methane production (Vivekanand et al. 2012) and normalized for production in IC. VS* volatile solids

*Spirochaetes*, *Bacteroidetes* and *Chloroflexi* were the dominant bacterial phyla in all three samples (Fig. 3a). Dominance of these phyla, with the exception of the *Spirochaetes*, is commonly observed in biogas processes (Nelson et al. 2011), whilst the low relative abundance of *Proteobacteria*- and *Firmicutes*-affiliated OTUs is in contrast with previous studies that have demonstrated their abundance in AD reactors (Kampmann et al. 2012; Nelson et al. 2011). The majority of the bacterial OTUs were distantly related to cultured relatives, whereas close similarities were observed with previous biogas microbial community studies describing uncultured phylotypes (Table 1). In particular, both BAC_nor-3 and BAC_nor-4 exhibited 99 % sequence identity to dominant *Chloroflexi*-affiliated OTUs that have been previously defined as highly prevalent core phylotypes involved in AD of sludge [Core group α-III and α-VI; (Rivière et al. 2009)]. The repeated detection of *Chloroflexi*-affiliated phylotypes in high abundance within biogas processes points towards a significant role and reveals a need for future investigations. Several OTUs of lower abundance demonstrated marked shifts depending on which substrate was present (Fig. 3b–c). BAC_nor-13, a *Petrobacter*-affiliated *betaproteobacteria* decreased approximately seven-fold in IC + SW digesters, whereas, the *Bacteroidales*-affiliated BAC_nor-12 and *Victivallis*-affiliated BAC_nor-26 experienced an eight-fold and nine-fold increase, respectively. The phenotype of BAC_nor-26 may be potentially interesting, as *Victivallis* sp. isolates have previously been described as capable of fermenting a variety of sugars including glucose and mannitol [found in brown seaweed; (Horn and Ostgaard 2001)] subsequently producing acetate, H_2_ and ethanol as end-products (Zoetendal et al. 2003).

**Fig. 3:**
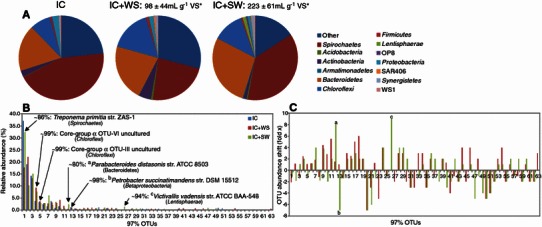
Relative abundance and comparison profiles of bacterial 16S rRNA OTUs identified in anaerobic digesters containing either waste water sludge with no additional organic substrate (inoculum, IC), IC plus wheat straw (IC + WS), or IC plus seaweed (IC + SW). Relative abundance of bacterial lineages at a phylum-level (**a)** and OTU-level (**b)** are shown. OTU abundance shifts between WS and SW digesters (**c)** were measured as either fold-change increases (+) or decreases (−) against IC measurements. Lineage information for selected OTUs (**a**–**c**) and OTU affiliation to previously described, highly prevalent core phylotypes (Rivière et al. 2009) is provided. Colour coding in **b** and **c** are as follows: IC, *red* indicates IC + WS and *green* indicates IC + SW. OTUs numbers in the *x*-axis corresponds to BAC_nor-terminology referred to in the text. Total methane yields are included in **a** for IC + WS and IC + SW, which are provided in the original publication on methane production (Vivekanand et al. 2012) and normalized for production in IC. VS* volatile solids

The present study shows that the microbial consortia involved in AD of seaweed comprise deeply branched OTUs. However, there are indications that trends in AD microbial profiles are beginning to emerge with the detection of several previously identified core group archaeal and bacterial phylotypes (Table 1; Rivière et al. 2009). Compared to the IC + WS digester, the IC + SW digester showed some conspicuous differences, the most prominent being an increase in methane production and the relative abundance of the *Methanosaeta concilii*-affiliated (presumably acetoclastic) ARC_nor-1. Given that methanogens are believed to rely on syntrophic relationships with bacteria for key metabolites (i.e., acetate, H_2_/CO_2_), ARC_nor-1 dominance is conceivably linked to bacterial population shifts and/or changes in bacterial metabolism. Surprisingly, dominant bacterial populations showed little variation between the digesters with larger shifts only observed for several low-abundant OTUs. Regardless, the large phylogenetic variation between biogas-producing communities and cultured representatives makes drawing definitive functional or interactive conclusions, a significant challenge. The functioning of biogas-producing microbial communities on the whole is insufficiently explored and requires further in depth structure–function analysis involving a combination of cultivation directed strategies and “meta-omic” approaches (i.e., metagenomics, metatranscriptomics).
